# The Elias University Hospital Approach: A Visual Guide to Ultrasound-Guided Botulinum Toxin Injection in Spasticity: Part III—Proximal Lower Limb Muscles

**DOI:** 10.3390/toxins17050240

**Published:** 2025-05-13

**Authors:** Marius Nicolae Popescu, Claudiu Căpeț, Cristina Beiu, Mihai Berteanu

**Affiliations:** 1 Department of Physical and Rehabilitation Medicine, Elias Emergency University Hospital, Carol Davila University of Medicine and Pharmacy, 020021 Bucharest, Romania; marius.popescu@umfcd.ro (M.N.P.); mberteanu@gmail.com (M.B.); 2 Clinic of Physical and Rehabilitation Medicine, Elias Emergency University Hospital, 011461 Bucharest, Romania; claudiu.capet@gmail.com; 3 Department of Oncologic Dermatology, Elias Emergency University Hospital, Carol Davila University of Medicine and Pharmacy, 020021 Bucharest, Romania

**Keywords:** post-stroke spasticity, botulinum toxin-A injections, ultrasound-guided therapy, proximal lower limb muscles, musculoskeletal ultrasound

## Abstract

Ultrasound-guided botulinum toxin type A (BoNT-A) injections have become an essential tool in the management of lower limb spasticity. Following our previous work, which focused on upper limb muscles, this third part provides a detailed visual guide to the identification and injection of proximal lower limb muscles frequently involved in spastic gait and posture disorders. This guide presents the ultrasound anatomy, clinical relevance, and injection strategies for eleven key muscles: *gluteus* *maximus*, *piriformis*, *psoas major*, *rectus* *femoris*, *sartorius*, *gracilis*, *adductor* *longus*, *adductor* *magnus*, *semimembranosus*, *semitendinosus*, and *biceps* *femoris*. For each muscle, the Elias University Hospital (EUH) model is applied, highlighting the zones of maximum thickness and motor point density to ensure precise and effective BoNT-A delivery. Enhanced with high-resolution ultrasound images and dynamic scanning techniques, this visual guide supports clinicians in performing safe, targeted injections. It serves as both an educational and practical reference for the ultrasound-guided treatment of spasticity in the proximal lower limb, completing the series and offering a standardized framework for comprehensive BoNT-A management. By promoting accurate toxin delivery, this approach is expected to improve functional mobility, reduce spasticity-related complications, and optimize patient-centered outcomes in rehabilitation settings.

## 1. Introduction

Lower limb spasticity is a major contributor to impaired posture, mobility, and gait in patients with upper motor neuron syndromes, including those resulting from stroke, cerebral palsy, spinal cord injury, or traumatic brain injury. It affects over 30% of stroke survivors and a significant proportion of individuals with cerebral palsy or traumatic brain injuries, contributing substantially to long-term disability and reduced mobility, which underscores the clinical importance of targeted interventions like BoNT-A [1]. Addressing focal muscle overactivity through targeted botulinum toxin type A (BoNT-A) injections has become a central component of personalized rehabilitation strategies, aiming to improve functional outcomes and enhance quality of life [2,3].

In our previous work, we provided a comprehensive guide to ultrasound-guided BoNT-A injections for upper limb muscles [4]. With this paper, we now shift focus to the lower limb, specifically targeting its proximal muscles. These muscles play a critical role in postural control, gait stability, and pelvic–lumbar alignment and are commonly involved in spastic gait patterns such as stiff knee gait, excessive knee flexion, hip retraction, or impaired limb advancement [5].

This guide presents a structured, ultrasound-based approach for identifying and injecting key proximal lower limb muscles, including the *gluteus maximus*, *piriformis*, *psoas major, rectus femoris*, *sartorius*, *gracilis*, *adductor longus*, *adductor magnus*, *semimembranosus*, *semitendinosus*, and *biceps femoris*. For each muscle, we describe its clinical relevance, scanning landmarks, dynamic features, zones of intramuscular neural arborization, and ultrasound-guided injection techniques, combining the literature-based knowledge with the practical insights gained from our daily work using the Elias University Hospital (EUH) model.

To enhance the practical value of this guide, we include high-resolution ultrasound images, clinical diagrams, and step-by-step video demonstrations. Together, these resources support clinicians in refining their technique and promoting standardized, evidence-based approaches to BoNT-A administration in spasticity management.

Among existing approaches to ultrasound-guided botulinum toxin injection in spasticity, the EUROMUSCULUS/USPRM Spasticity Approach stands out for offering a concise overview of target muscles in the lower limb [6]. However, it includes only a limited number of schematic illustrations and lacks dynamic ultrasound guidance or clinical photographic references. In contrast, the Elias University Hospital (EUH) model introduced in this series aims to fill that gap by offering a systematic, muscle-by-muscle visual approach. Each target muscle is presented with clinical positioning photos, high-resolution ultrasound images, key anatomical landmarks, and dynamic scanning guidance, creating a reproducible and educational model suited to real-world clinical applications and training.

## 2. General Methodology

In our clinical practice, ultrasound-guided botulinum toxin injections were performed using a GE Venue Go system equipped with a linear 12 L transducer. A 21 G needle was used for all injections.

Patient positioning was adapted based on the anatomical region being evaluated. In most cases, the standard position can be inferred from the clinical photographs provided alongside the corresponding ultrasound images. Specific positioning details are explicitly described in cases requiring non-standard setups—for instance, supine positioning with hip abduction and external rotation for injections targeting the adductor magnus and gracilis muscles.

## 3. Proximal Lower Limb Muscles Implicated in Post-Stroke Spasticity

### 3.1. Gluteus Maximus (Gmax)

#### 3.1.1. Overview

The *gluteus maximus* (Gmax) is a muscle targeted in the spastic patterns of the lower limb that involve resistance to passive movement during hip flexion and the internal rotation of the thigh at the hip joint [7,8,9].

#### 3.1.2. Ultrasound Identification

In our clinical practice, the Gmax can be identified via musculoskeletal ultrasound by placing the transducer at a 45-degree angle toward the sacrum on the posterior surface of the hip joint, in the upper quadrant of the gluteal region, approximately 8 cm lateral to the sacrum. Superficial to the iliac cortex, three muscle masses can be visualized: the *gluteus minimus* (deepest), the *gluteus medius*, and the *gluteus maximus* (most superficial).

#### 3.1.3. Key Ultrasound Landmarks

**Figure 1:**
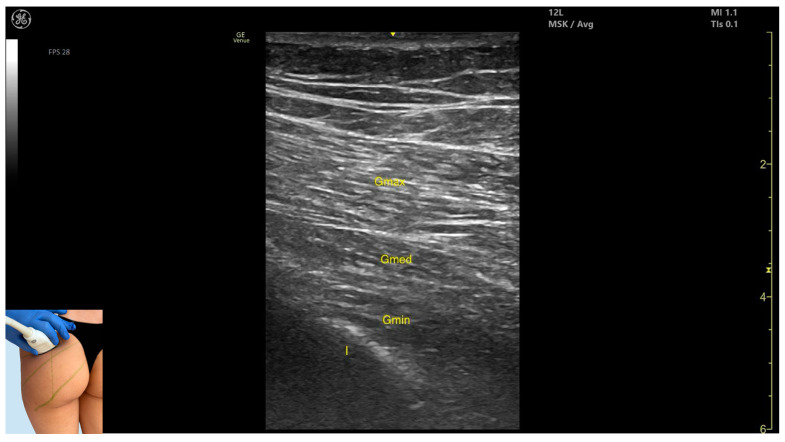
Ultrasound anatomy of *gluteus maximus* (Gmax) with key landmarks: Gmax—*gluteus maximus*; Gmed—*gluteus medius*; Gmin—*gluteus minimus*; I—iliac bone.

The key ultrasound landmarks include the following (Figure 1) [7,8,9]:

- Muscle morphology: It is the strongest and most superficial muscle in the gluteal group.
- Muscle position: It represents the most superficial muscle mass at this level. It lies superficial to both the *gluteus medius* and *gluteus minimus*.
- External fascia: It is enclosed by a pronounced fascia, which separates it from the subcutaneous tissue and the *gluteus medius*, facilitating botulinum toxin injection.
- Dynamic evaluation: During dynamic evaluation, scanning laterally toward the hip joint reveals an increase in muscle thickness of the *gluteus medius* and *gluteus minimus*. Muscle contraction is visible during hip extension and external rotation maneuvers.

#### 3.1.4. Clinical Implications and Strategy

The Gmax is involved in spastic patterns that limit hip flexion and impair swing limb advancement during gait [10].

Studies have shown that BoNT-A injections into the Gmax not only reduce spasticity but also improve ulcerative lesions in the gluteal and intergluteal regions [10,11,12].

In cases of localized contracture following total hip arthroplasty, BoNT-A injections into the *gluteus maximus* have led to complete pain relief and contributed to gait improvement during rehabilitation [13,14].

A 2024 study aimed at identifying the zones with the highest intramuscular neural arborization divided the *gluteus maximus* using three reference lines [15]:

- Line (a): from the posterior superior iliac spine (PSIS) to the greater trochanter (GT) of the femur;
- Line (c): from the coccyx (CO) to the ischial tuberosity (IT);
- Line (b): drawn midway between lines a and c.

The areas with the highest nerve arborization were found at 40–70% along the cranial segment (line a), 30–60% along the middle segment (line b), and 40–70% along the caudal segment (line c).

These findings suggest the need for three injection points when targeting this muscle [15].

In our clinical practice, the preferred injection sites for BoNT-A are determined by maximum muscle thickness identified via ultrasound:

For the cranial segment, the transducer is positioned at a 45-degree angle toward the sacrum on the posterior surface of the hip joint, in the upper quadrant of the gluteal region, approximately 8 cm lateral to the sacrum.

From this position, scanning is continued distally along the posterior thigh, with injections administered to the middle and caudal segments of the *gluteus maximus*.

### 3.2. Piriformis

#### 3.2.1. Overview

The *piriformis* is a muscle targeted in the spastic patterns of the lower limb that involve resistance to passive movement during the internal rotation and adduction of the thigh at the hip joint [16,17].

#### 3.2.2. Ultrasound Identification

In our clinical practice, the *piriformis* muscle can be identified using musculoskeletal ultrasound by placing the transducer transversely on the posterior surface of the hip joint, angled approximately 20 degrees laterally toward the sacrum, about 8 cm lateral to the sacrum and 10 cm proximal to the ischial tuberosity. From deep to superficial, the following structures can be visualized: the acetabular cortex, the *piriformis* muscle, and the *gluteus maximus*, which lies superficial to the *piriformis*.

#### 3.2.3. Key Ultrasound Landmarks

**Figure 2:**
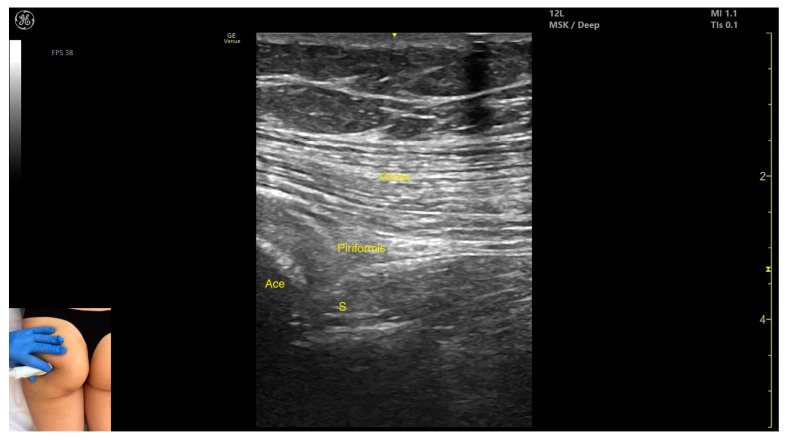
Ultrasound anatomy of *piriformis* with key landmarks: Gmax—*gluteus maximus*; Ace—acetabulum; S—sciatic nerve.

The key ultrasound features include the following (Figure 2) [16,17]:

- Muscle morphology: It is part of the deep gluteal muscle group, along with the *obturator internus*, *obturatorus externus*, *superior gemellus*, *inferior gemellus*, and *quadratus femoris*.
- Muscle position: At this level, it appears as the first muscle mass superficial to the acetabular cortex. The sciatic nerve lies in close proximity, located deep to the *piriformis*.
- External fascia: It presents a well-defined fascia separating it from the *gluteus maximus*, which is important during botulinum toxin injection.
- Dynamic evaluation: During dynamic evaluation, scanning 2–3 cm distally toward the ischial tuberosity, the *piriformis* muscle tapers and disappears, and the *obturator internus* muscle becomes visible. Superficial to the *obturator internus*, the *gluteus maximus* and the sciatic nerve (within the intermuscular fascia) can be observed. Muscle contraction is visible during the external rotation and abduction of the femur at the hip joint.

#### 3.2.4. Clinical Implications and Injection Strategy

BoNT-A injections into the *piriformis* muscle are used both to reduce muscle spasms and to relieve pain in piriformis syndrome, a condition where the muscle compresses the sciatic nerve [18,19,20,21].

The region with the highest intramuscular nerve arborization has been identified between three-fifths and four-fifths of the distance from the greater trochanter to the sacrum, along the muscle [22].

In our clinical practice, the preferred injection site for BoNT-A is the point of maximum muscle thickness, identified via ultrasound, with the transducer placed transversely on the posterior surface of the hip joint, angled approximately 20 degrees toward the sacrum, about 8 cm lateral to the sacrum and 10 cm proximal to the ischial tuberosity.

The use of musculoskeletal ultrasound in approaching the *piriformis* allows for the accurate targeting of the injection site and prevents nerve injury, especially to the nearby sciatic nerve.

### 3.3. Psoas Major

#### 3.3.1. Overview

The *psoas major* is a muscle targeted in the spastic patterns of the lower limb that involve resistance to passive movement during hip extension and extension or medial inflection at the lumbar spine [8,23].

#### 3.3.2. Ultrasound Identification

In our clinical practice, the *psoas major* can be identified using musculoskeletal ultrasound by placing the transducer transversely on the posterior surface of the trunk, approximately 2 cm lateral to the spinous process of the L4 vertebra and 1 cm proximal to the iliac crest. Lateral to the L4 spinous process, the following structures are observed, from superficial to deep: the *erector spinae* muscles, immediately adjacent to the spinous process, the *quadratus lumborum*, lateral and superficial to the *psoas*, the *psoas major*, located deeper, and the intraperitoneal space, situated deep to the *psoas*.

#### 3.3.3. Key Ultrasound Landmarks

**Figure 3:**
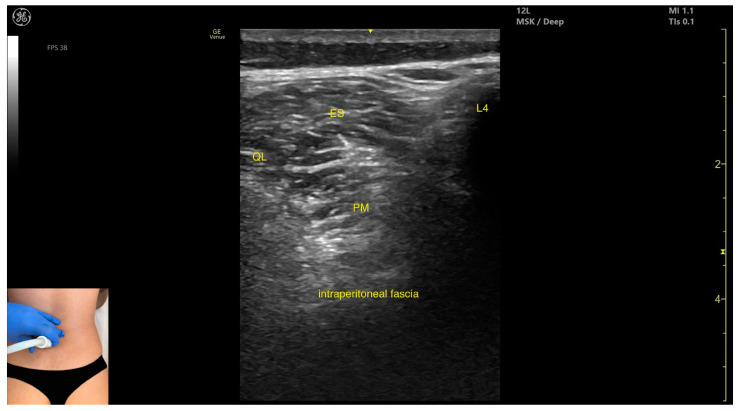
Ultrasound anatomy of *psoas major* (PM) (with the transducer transversely) with key landmarks: PM—*psoas major*; L4—spinous process of the L4 vertebra; ES—*erector spinae*; QL—*quadratus lumborum*.

**Figure 4:**
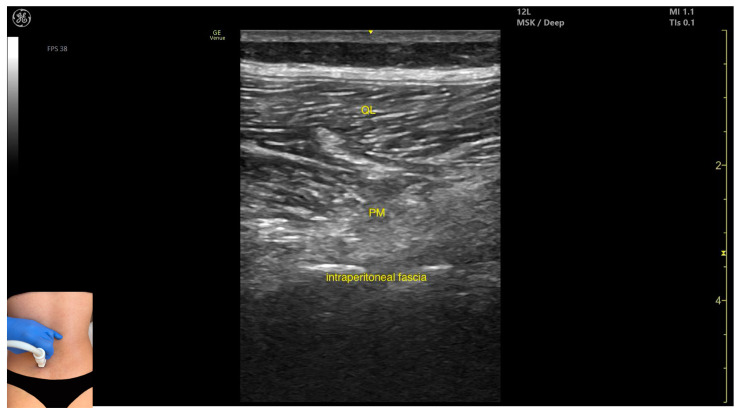
Ultrasound anatomy of *psoas major* (PM) (with the transducer longitudinally) with key landmarks: QL—*quadratus lumborum*; PM—psoas major.

The key ultrasound features include the following (Figure 3 and Figure 4) [7,8,23]:

- Muscle position: It is the deepest muscle mass at this level.
- External fascia: It presents a pronounced fascia that separates it from the *erector spinae* and *quadratus lumborum*, aiding in safe botulinum toxin injection.
- Dynamic evaluation: During dynamic evaluation, when scanning proximo-medially toward the costal arch, its origin is visualized from the T12 to L5 vertebrae.
- Contraction is visible during hip flexion, as well as lumbar spine flexion and lateral inflection maneuvers.
- The *psoas major* can also be visualized using a longitudinal transducer position, placed superior to the iliac crest and 2 cm lateral to the L4 spinous process. In this view, both the *psoas major* and the overlying *quadratus lumborum* are seen superficial to the intraperitoneal space.

#### 3.3.4. Clinical Implications and Injection Strategy

In ambulatory patients, the *psoas* muscle contributes to spastic patterns that impede physiological hip extension during the mid-stance and terminal stance phases of gait.

In non-ambulatory patients, the spasticity of the *psoas* muscle leads to incorrect sitting posture and myotendinous retractions while seated in a wheelchair, which can also complicate nursing care [24].

The region with the highest intramuscular nerve arborization is located at approximately 50% along the reference line drawn from the T12 vertebra to the midpoint of the inguinal ligament [25].

In our clinical practice, the preferred injection site for BoNT-A is at the point of maximum muscle thickness identified via ultrasound, using either a transverse or longitudinal transducer orientation on the posterior trunk, approximately 2 cm lateral to the L4 spinous process and 1 cm proximal to the iliac crest.

The use of musculoskeletal ultrasound in approaching the *psoas major* allows for the precise targeting of the muscle while avoiding the intraperitoneal space.

### 3.4. Rectus Femoris (RF)

#### 3.4.1. Overview

The *rectus femoris* (RF) is a muscle frequently targeted in the spastic patterns of the lower limb that involve resistance to passive movement during hip extension and knee flexion maneuvers [16].

#### 3.4.2. Ultrasound Identification

In our clinical practice, the RF can be identified using musculoskeletal ultrasound with the transducer placed transversely at the proximal third of the anterior thigh, along the midline. Superficial to the femoral cortex, two muscle masses are visualized: the vastus intermedius (deep) and the *rectus femoris* (superficial and oval-shaped).

#### 3.4.3. Key Ultrasound Landmarks

**Figure 5:**
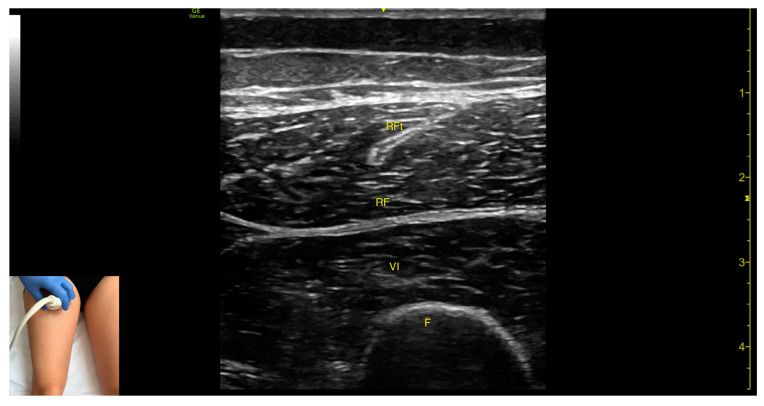
Ultrasound anatomy of *rectus femoris* (RF) with key landmarks: RF—*rectus femoris*; RFt—*rectus femoris* tendon; VI—vastus intermedius; F—femur.

The key ultrasound features include the following (Figure 5) [8,16]:

- Muscle morphology: The RF is a bipennate muscle, characterized by a central septum, with muscle fibers attached on both sides and continuing into a central tendon. These two fiber regions should be individually targeted during injection
- Muscle position: It is the most superficial muscle mass at this level. Lateral to the *rectus femoris* lies the vastus lateralis, and medial to it, the vastus medialis.
- External fascia: A pronounced fascia separates the *rectus femoris* from the subcutaneous plane and adjacent muscles: vastus intermedius, vastus lateralis, and vastus medialis.
- Dynamic evaluation: During dynamic evaluation, scanning distally toward the knee joint, the muscle bulk of the RF decreases, but its central tendon remains visible until the distal third of the thigh (Video S1). Muscle contraction is visible during hip flexion and knee extension maneuvers.

#### 3.4.4. Clinical Implications and Injection Strategy

The RF is involved in the stiff knee gait spastic pattern. According to the literature, BoNT-A injections in this muscle can reduce spasticity and improve gait pattern [26,27,28,29,30].

In patients with spastic hemiparesis, studies have shown that BoNT-A injections into the RF result in improved muscular kinematics during gait, including greater maximal muscle lengthening and improved muscle stretch capacity [31,32].

The zones with the highest concentration of motor points in the RF are located at approximately 40% and 60% along a reference line connecting the anterior superior iliac spine (ASIS) to the superior edge of the patella [33,34].

In our clinical practice, the preferred injection sites are the areas of maximum muscle thickness, identified via ultrasound, with the transducer placed transversely approximately 2 cm proximal and 2 cm distal to the mid-thigh level on the anterior midline.

### 3.5. Sartorius (Sart)

#### 3.5.1. Overview

The *sartorius* (Sart) is a muscle targeted in the spastic patterns of the lower limb that involve resistance to passive movement during hip extension, adduction, and internal rotation, as well as knee extension [23].

#### 3.5.2. Ultrasound Identification

In our clinical practice, the Sart can be identified using musculoskeletal ultrasound with the transducer placed transversely on the middle third of the medial thigh. Superficial to the femoral cortex, the *adductor magnus* is visualized, over which lie the *adductor longus* and vastus medialis, and the most superficial structure is the Sart muscle.

#### 3.5.3. Key Ultrasound Landmarks

**Figure 6:**
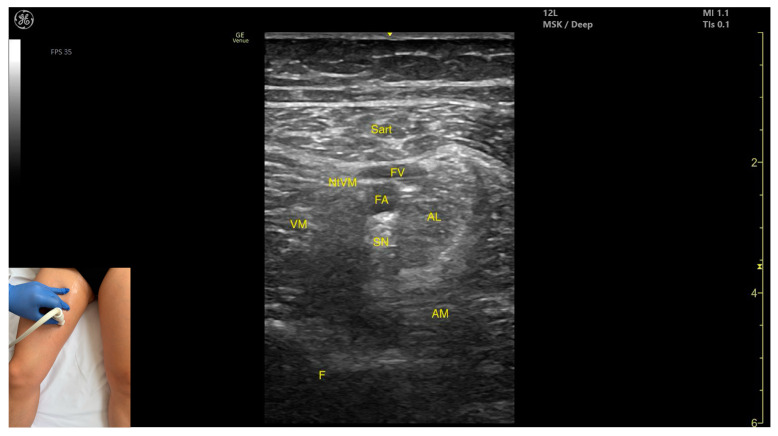
Ultrasound anatomy of *sartorius* (Sart) with key landmarks: Sart—sartorius; FV—femoral vein; FA—femoral artery; SN—saphenous nerve; NtVM—nerve to vastus medialis; AL—*adductor longus*; VM—vastus medialis; AM—*adductor magnus*; F—femur.

The key ultrasound features include the following (Figure 6) [16,23,34,35]:

- Muscle position: It is the most superficial muscle in the anterior compartment of the thigh. Deep and lateral to the *sartorius* lies the vastus medialis; deep and medial to the *sartorius* lies the *adductor longus*. At this level, the adductor canal is visualized beneath the Sart, containing the femoral artery, femoral vein, saphenous nerve, and nerve to vastus medialis.
- External fascia: A pronounced fascia separates the Sart from the subcutaneous plane, vastus medialis, *adductor longus*, and *adductor magnus*, aiding in precise botulinum toxin injection.
- Dynamic evaluation: During dynamic evaluation, scanning proximally toward the hip joint, at the proximal third of the anterior thigh, the adductor longus thickens medial and deep to the Sart, while the *rectus femoris* appears deep and lateral. At this level, the adductor canal shifts to lie medial to the Sart and lateral to the *adductor longus*. During distal scanning toward the knee joint, in the distal third of the anterior thigh, the *gracilis* is seen medial to the Sart, and the vastus medialis lies deep and lateral to it. Muscle contraction is visible during hip flexion, abduction, and internal rotation, as well as knee flexion maneuvers.

#### 3.5.4. Clinical Implications and Injection Strategy

The zones with the highest intramuscular nerve arborization in the Sart are located at 20–40% and 60–80% along a reference line from the most prominent point of the anterior superior iliac spine (0%) to the medial femoral condyle (100%) [36].

In our clinical practice, the preferred injection sites are the areas of maximum muscle thickness, identified via ultrasound, with the transducer placed transversely on the proximal third of the anterior medial thigh and on the distal third of the anterior medial thigh.

The use of musculoskeletal ultrasound for targeting the Sart muscle allows for the precise delivery of the injection and helps to avoid neurovascular injury.

### 3.6. Gracilis (G)

#### 3.6.1. Overview

The *gracilis* (G) muscle is commonly targeted in spastic patterns involving resistance to passive movement during hip abduction, knee extension, and the external rotation of the leg [8,16].

#### 3.6.2. Ultrasound Identification

In our clinical practice, the G can be identified using musculoskeletal ultrasound with the lower limb positioned in supine, with the hip abducted, externally rotated, and semi-flexed, and the knee in semi-flexion. The transducer is placed transversely on the proximal third of the medial thigh. Superficial to the femoral cortex, two muscle masses are visualized: the *adductor magnus* and the *semimembranosus*, and the *gracilis* lies superficial to both.

#### 3.6.3. Key Ultrasound Landmarks

**Figure 7:**
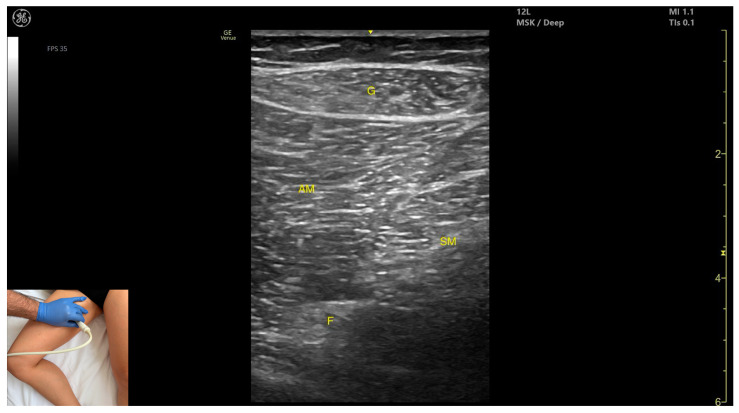
Ultrasound anatomy of *gracilis* (G) with key landmarks: G—gracilis; AM—*adductor magnus*; SM—*semimembranosus*; F—femur.

The key ultrasound landmarks include the following (Figure 7) [8,16,23]:

- Muscle position: It is the most superficial and medial muscle mass of the thigh. Lateral and deep to the G lies the *adductor longus*, while medial and deep to the G lies the *semimembranosus*.
- External fascia: It presents a well-defined fascia that separates it from the subcutaneous plane and from the *adductor magnus*, *adductor longus*, and *semimembranosus*, which is relevant for BoNT-A injections.
- Dynamic evaluation: During dynamic evaluation, scanning toward the distal third of the medial thigh, the following are observed: the relationship between the G and *adductor magnus* remains consistent, the *adductor longus* disappears from view, the Sart muscle appears, joining the *gracilis*, and the adductor canal becomes visible, located deep and lateral to the *gracilis*. In the distal third of the medial thigh, the G is positioned lateral to the *semitendinosus*. Scanning further distally beyond the knee joint, the tendons of the G, *sartorius*, and *semitendinosus* converge to form the pes anserinus on the medial aspect of the proximal tibia. Muscle contraction is visible during hip adduction, knee flexion, and internal rotation maneuvers.

#### 3.6.4. Clinical Implications and Injection Strategy

Reducing spasticity in the gracilis muscle through BoNT-A injection can lead to several clinically meaningful improvements. These include enhanced hip abduction and knee extension, reduced scissoring during gait, improved hygiene and seating posture, and better tolerance for orthotic devices. In pediatric patients with cerebral palsy, such injections may also prevent hip dislocation and delay or reduce gait deterioration [36]. Overall, this intervention contributes to improved mobility, functional independence, and quality of life.

According to a study by Won et al., the area with the highest intramuscular nerve arborization of the *gracilis* is located between 25 and 35% along a reference line drawn from the pubic tubercle to the line connecting the center of the patella and the medial femoral condyle [37,38].

In our clinical practice, the preferred injection site for BoNT-A is the area of maximum muscle thickness, identified via ultrasound, with the transducer placed transversely on the proximal third of the medial thigh.

### 3.7. Adductor Longus (AL)

#### 3.7.1. Overview

The *adductor longus* is a muscle targeted in the spastic patterns of the lower limb that involve resistance to passive movement during hip abduction and the external rotation of the thigh [16].

#### 3.7.2. Ultrasound Identification

In our clinical practice, the *adductor longus* can be identified using musculoskeletal ultrasound by placing the transducer transversely on the proximal third of the medial thigh. From deep to superficial, the following structures are visualized: the femoral cortex and three muscle layers: *adductor magnus*, *adductor brevis*, and *adductor longus*.

#### 3.7.3. Key Ultrasound Landmarks

**Figure 8:**
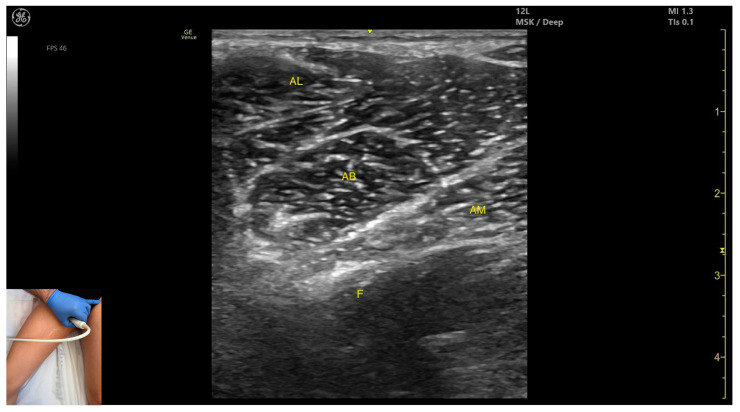
Ultrasound anatomy of *adductor longus* (AL) with key landmarks: AL—*adductor longus*; AB—*adductor brevis*; AM—*adductor magnus*; F—femur.

The key ultrasound landmarks include the following (Figure 8) [16]:

- Muscle position: It is the most superficial muscle mass at this level. Lateral to it lies the *sartorius* muscle, and medially, the *gracilis* muscle. The adductor canal is also visualized laterally.
- External fascia: It presents a pronounced fascia that separates it from the *adductor magnus*, *adductor brevis*, *sartorius*, and *gracilis* muscles during botulinum toxin injection.
- Dynamic evaluation: During dynamic evaluation, scanning proximally toward the hip joint, the *adductor brevis*, which lies deep to the *adductor longus*, becomes more prominent. Scanning distally toward the distal third of the medial thigh, the adductor longus gradually decreases in thickness until it disappears from view, at which point the *gracilis* and *sartorius* muscles become adjacent (Video S2). Contraction is visible during hip adduction and internal rotation maneuvers.

#### 3.7.4. Clinical Implications and Injection Strategy

The region with the highest density of motor points in the *adductor longus* is located at 40–50% of the muscle length, corresponding to approximately three-fifths of the muscle’s total length [39].

In our clinical practice, the preferred injection site for BoNT-A is at the point of maximum muscle thickness, identified via ultrasound, with the transducer placed transversely on the proximal third of the medial thigh.

The use of musculoskeletal ultrasound in targeting the *adductor longus* allows for the precise placement of the toxin while avoiding neurovascular injury.

### 3.8. Adductor Magnus (AM)

#### 3.8.1. Overview

The *adductor magnus* (AM) is a muscle targeted in the spastic patterns of the lower limb that involve resistance to passive movement during hip abduction and hip extension [2].

#### 3.8.2. Ultrasound Identification

In our clinical practice, the AM can be identified using musculoskeletal ultrasound with the lower limb positioned supine, the hip abducted, externally rotated, and in semi-flexion, and the knee also in semi-flexion. The transducer is placed transversely on the proximal third of the medial thigh. Superficial to the femoral cortex, two muscle masses are visualized: the AM and the *semimembranosus*, and the *gracilis* lies superficial to both.

#### 3.8.3. Key Ultrasound Landmarks

**Figure 9:**
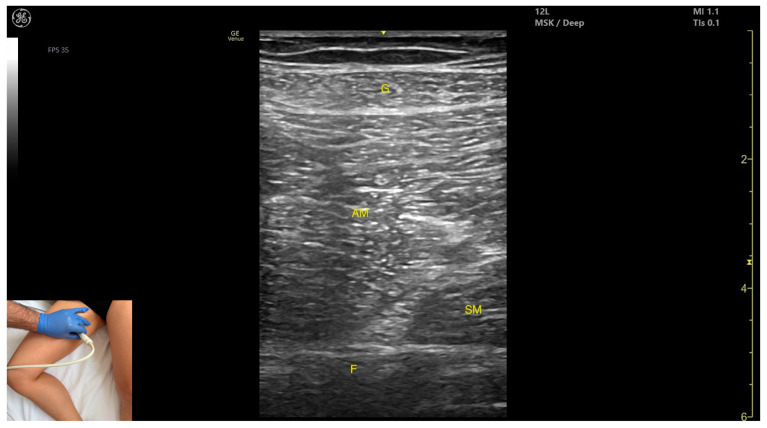
Ultrasound anatomy of adductor magnus (AM) with key landmarks: G—*gracilis*; AM—*adductor magnus*; SM—*semimembranosus*; F—femur.

The key ultrasound landmarks include the following (Figure 9) [8]:

- Muscle morphology: It is the largest and deepest muscle of the adductor group.
- Muscle position: It is the first muscle mass superficial to the femoral cortex at this level. It is covered superficially by the *gracilis* muscle; medial to it lies the *semimembranosus*.
- External fascia: It has a well-defined fascia separating it from the *gracilis* and *semimembranosus*, supporting safe BoNT-A injections.
- Dynamic evaluation: During dynamic evaluation, scanning distally toward the knee joint, a reduction in muscle bulk is observed in both the AM and *gracilis*. Muscle contraction is visible during hip adduction and hip flexion maneuvers.

#### 3.8.4. Clinical Implications and Injection Strategy

In the pediatric population with cerebral palsy, BoNT-A injections into the AM have been shown to significantly reduce spasticity and help prevent hip dislocation [40].

According to a study by Santamato et al., BoNT-A injections into the AM following total hip arthroplasty led to a significant reduction in pain—as measured on the Visual Analog Scale (VAS)—and contributed to gait improvement during rehabilitation [41].

The area with the highest concentration of motor points in the AM is located at 40% along a reference line drawn from the pubic symphysis to the medial knee joint line, corresponding to the middle third of the thigh [42].

In our clinical practice, the preferred injection site for BoNT-A is at the point of maximum muscle thickness, identified via ultrasound, with the transducer placed transversely on the middle third of the medial thigh.

### 3.9. Semimembranosus (SM)

#### 3.9.1. Overview

The *semimembranosus* (SM) is a muscle targeted in the spastic patterns of the lower limb that involve resistance to passive movement during hip flexion, knee extension, and the external rotation of the leg [8,16].

#### 3.9.2. Ultrasound Identification

In our clinical practice, the SM can be identified using musculoskeletal ultrasound with the transducer placed transversely on the distal third of the posterior thigh, in the medial portion. Superficial to the femoral cortex, the adductor magnus is visualized, and superficial to it, two muscle masses appear: SM (medial) and *semitendinosus* (lateral).

#### 3.9.3. Key Ultrasound Landmarks

**Figure 10:**
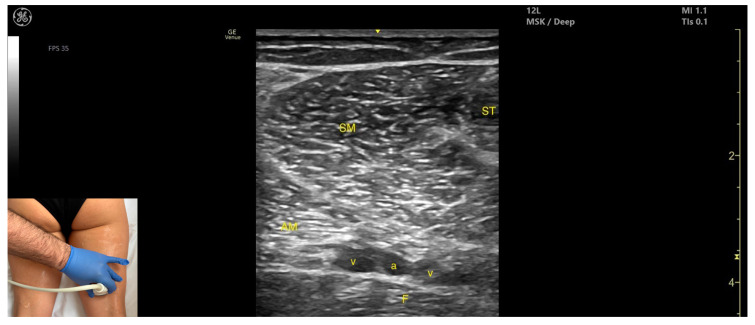
Ultrasound anatomy of *semimembranosus* (SM) with key landmarks: SM—*semimembranosus*; ST—*semitendinosus*; AM—*adductor magnus*; v—vein, a—artery, F—femur.

The key ultrasound features include the following (Figure 10) [8,16,43]:

- Muscle position: It is a superficial muscle mass at this level. It is the first muscle seen from medial to lateral on the posterior surface of the thigh. Deep and medial to it lies the *adductor magnus*, while laterally to it lie the *semitendinosus* and *biceps femoris* muscles. The sciatic nerve is located deep and lateral to the SM and *semitendinosus* and deep to the *biceps femoris*. The popliteal vascular bundle is visualized deep to the muscle in the distal third of the posterior thigh.
- External fascia: It has a pronounced fascia that separates it from the subcutaneous tissue, *semitendinosus*, and *adductor magnus*, aiding in safe botulinum toxin injection.
- Dynamic evaluation: During dynamic evaluation, scanning proximally toward the hip joint, the SM decreases in thickness, while the *semitendinosus* increases; the sciatic nerve remains lateral and deep to the *semitendinosus*. Contraction is visible during hip extension, knee flexion, and the internal rotation of the leg.

#### 3.9.4. Clinical Implications and Injection Strategy

The SM contributes to excessive knee flexion during gait in patients with spasticity [5].

BoNT-A injections into this muscle can improve gait mechanics by enhancing knee extension, anterior pelvic tilt, and increasing the rate of muscle lengthening in the *semimembranosus* [44].

The region with the highest intramuscular nerve arborization is located at 20–40% along a reference line from the medial and lateral femoral condyles (0%) to the ischial tuberosity (100%) [45].

In our clinical practice, the preferred injection site for BoNT-A is the point of maximum muscle thickness, identified via ultrasound, with the transducer placed transversely on the distal third of the posterior medial thigh.

The use of musculoskeletal ultrasound for targeting the SM ensures the accurate delivery of the substance while minimizing the risk of neurovascular injury.

### 3.10. Semitendinosus (ST)

#### 3.10.1. Overview

The *semitendinosus* (ST) is a muscle commonly targeted in the spastic patterns of the lower limb that involve resistance to passive movement during hip flexion, knee extension, and the external rotation of the leg [8,16].

#### 3.10.2. Ultrasound Identification

In our clinical practice, the ST can be identified using musculoskeletal ultrasound with the transducer placed transversely on the proximal third of the posterior thigh, in the medial portion. Superficial to the femoral cortex, three muscle masses are observed: the *adductor magnus*, the *semimembranosus* (medial), and the ST (lateral).

#### 3.10.3. Key Ultrasound Landmarks

**Figure 11:**
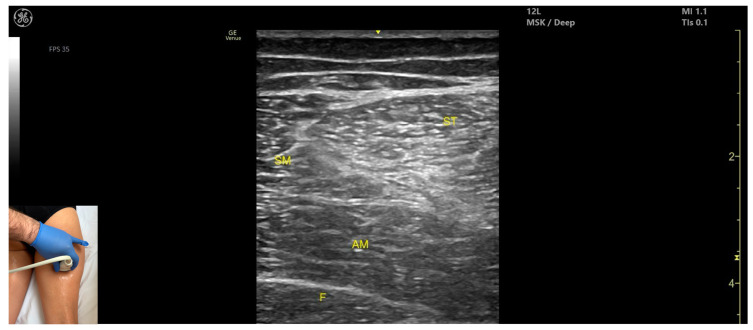
Ultrasound anatomy of *semitendinosus* (ST) with key landmarks: ST—*semitendinosus*; SM—*semimembranosus*; AM—*adductor magnus*; F—femur.

**Figure 12:**
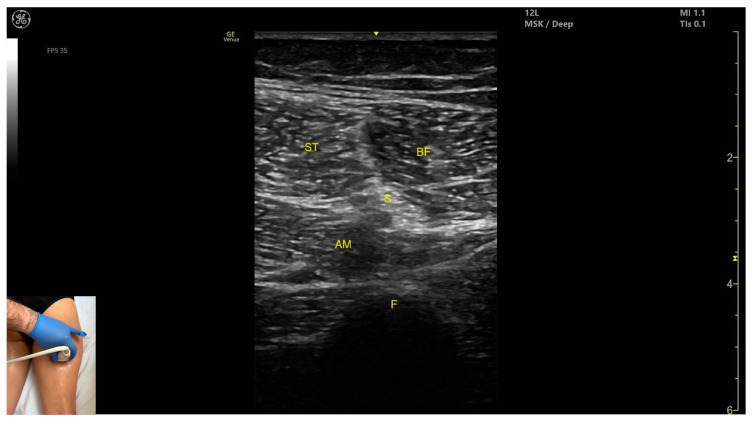
Mercedes-Benz sign: ST—*semitendinosus*; BF—*biceps femoris*, S—sciatic nerve; AM—*adductor magnus*; F—femur.

The key ultrasound features include the following (Figure 11 and Figure 12) [43,46,47,48,49]:

- Muscle position: It appears as a superficial muscle mass at this level. It is the second muscle from medial to lateral in the proximal third of the posterior thigh. It is bordered medially by the *semimembranosus* and laterally by the *biceps femoris*. The sciatic nerve lies deep and lateral to the ST and deep to the *biceps femoris*.
- Muscle morphology: The “Mercedes-Benz sign” may be seen—an ultrasound pattern created by the intersection of the fascial planes of the ST, *biceps femoris*, and *adductor magnus*, with the sciatic nerve centered between them. A characteristic “Venetian blind” appearance can be observed—this refers to the vertical intramuscular fascia traversing the muscle’s width. It appears as alternating hyperechoic and hypoechoic bands resembling the slats of a Venetian blind, resulting from the regular alignment of muscle fibers and connective septa reflecting ultrasound waves (Video S3).
- External fascia: It features a pronounced fascia that separates it from the subcutaneous layer, *adductor magnus*, *semimembranosus*, and *biceps femoris*, which is crucial for safe BoNT-A injection.
- Dynamic evaluation: During dynamic scanning distally toward the knee joint, the ST decreases in thickness, while the *semimembranosus* increases. The sciatic nerve maintains its course deep and lateral to the *semitendinosus*. Muscle contraction is visible during hip extension, knee flexion, and the internal rotation of the leg.

#### 3.10.4. Clinical Implications and Injection Strategy

The ST contributes to excessive knee flexion during gait in patients with lower limb spasticity [5].

The areas with the highest intramuscular nerve arborization are located at 25–40% and 60–80% along a reference line drawn from the medial and lateral femoral condyles (0%) to the ischial tuberosity (100%) [45].

In our clinical practice, the preferred injection sites for BoNT-A are the regions of maximum muscle thickness, identified via ultrasound, with the transducer placed transversely on the proximal and distal thirds of the medial posterior thigh.

The use of musculoskeletal ultrasound in targeting the ST allows for precise placement and helps avoid nerve injury.

### 3.11. Biceps Femoris (BF)

#### 3.11.1. Overview

The *biceps femoris* (BF) is a muscle targeted in the spastic patterns of the lower limb that involve resistance to passive movement during hip flexion and internal rotation, as well as knee extension and the internal rotation of the leg [7,8,50].

#### 3.11.2. Ultrasound Identification

In our clinical practice, the BF can be identified using musculoskeletal ultrasound with the transducer placed transversely on the distal third of the posterior thigh, in the lateral portion. Superficial to the femoral cortex, the BF muscle is visualized.

#### 3.11.3. Key Ultrasound Landmarks

**Figure 13:**
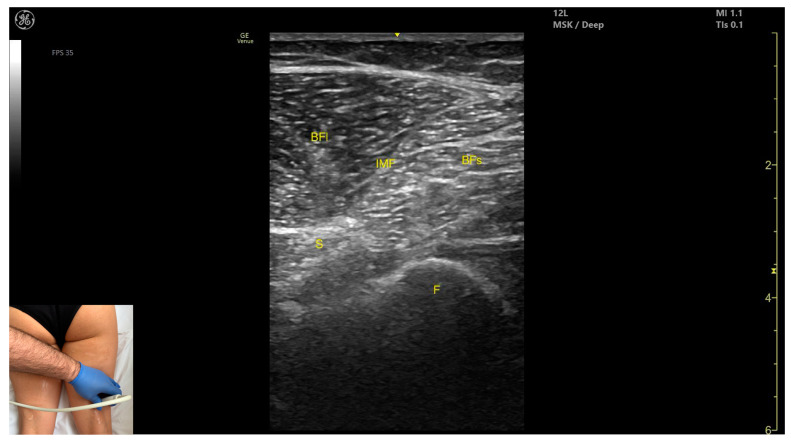
Ultrasound anatomy of *biceps femoris* (BF) with key landmarks: BFl—*biceps femoris* long head; IMF—intramuscular fascia; BFs—*biceps femoris* short head; S—sciatic nerve; F—femur.

The key ultrasound features include the following (Figure 13) [7,8,35,43,50]:

- Muscle morphology: It has two heads: the long head (medial) and the short head (lateral), which can be approached individually, separated by an intramuscular fascia.
- Muscle position: It appears as a superficial muscle mass and is the most lateral muscle in the posterior (flexor) compartment of the thigh at this level. The long head lies adjacent to the sciatic nerve, which runs underneath the muscle.
- External fascia: A pronounced fascia separates the BF from the subcutaneous plane, and from the vastus lateralis and *semitendinosus*, which is relevant during BoNT-A injections.
- Dynamic evaluation: During dynamic evaluation, scanning proximally toward the hip joint, the long head increases in thickness while the short head decreases. At the mid-thigh, the short head disappears, and the long head reaches its maximum thickness. Scanning distally toward the knee joint, the short head increases in thickness, and the long head decreases. Muscle contraction is visible during hip extension, external rotation, knee flexion, and the external rotation of the leg.

#### 3.11.4. Clinical Implications and Injection Strategy

The BF is implicated in spastic gait patterns characterized by excessive knee flexion during walking [5].

The highest density of intramuscular nerve arborization is located at the following:

- 15–30% of the reference line for the short head;
- 50–60% of the reference line for the long head.

This is measured along a line drawn from the medial and lateral femoral condyles (0%) to the ischial tuberosity (100%) [45].

In our clinical practice, the preferred injection sites for BoNT-A injections are the points of maximum muscle thickness, identified via ultrasound:

- For the short head, the transducer is placed transversely on the distal third of the posterior-lateral thigh;
- For the long head, the transducer is placed transversely at mid-thigh level on the posterior-lateral thigh.

The use of musculoskeletal ultrasound in targeting the BF allows for accurate substance placement while avoiding nerve injury.

Table 1 captures the key tips and precautions for BoNT-A injections in each lower limb muscle.

## 4. Conclusions

This paper is the third part of the series The Elias University Hospital Approach: A Visual Guide to Ultrasound-Guided Botulinum Toxin Injection in Spasticity and focuses on the practical application of musculoskeletal ultrasound for guiding BoNT-A injections into the proximal lower limb muscles in patients with post-stroke or neurologically induced spasticity.

By placing ultrasound at the core of the injection strategy, this guide provides clinicians with a reliable, evidence-based, and reproducible approach to safely target key muscles such as the *gluteus maximus*, *piriformis*, *psoas major*, *rectus femoris*, *sartorius*, *gracilis*, adductor longus, *adductor magnus*, *semimembranosus*, *semitendinosus*, and *biceps femoris*. For each muscle, we have described essential anatomical landmarks, dynamic sonographic features, neural arborization zones, and precise injection techniques informed by both the current literature and our clinical experience at Elias University Hospital.

Together with the previously published upper limb volumes, this paper contributes to a unified and standardized framework for ultrasound-guided spasticity management across major muscle groups. In the next part, we will continue with the lower limb, addressing distal muscles.

## Figures and Tables

**Table 1:** Key safety considerations for ultrasound-guided botulinum toxin type A (BoNT-A) injections into lower limb muscles.

Muscle	Key Anatomical Landmarks	Nearby Structures at Risk	Ultrasound Technique Tip	Injection Precautions
Gluteus Maximus	Upper gluteal region; 8 cm lateral to sacrum	Gluteus medius and minimus (deep); sciatic nerve (potentially)	Scan angled at 45° toward the sacrum	Avoid sciatic nerve injury
Piriformis	8 cm lateral to sacrum; 10 cm proximal to the ischial tuberosity	Gluteus maximus (superficial); sciatic nerve (deep to muscle)	Transverse scan angled ~20° laterally toward the sacrum	Visualize piriformis and sciatic nerve; avoid nerve injury
Psoas Major	2 cm lateral to L4 spinous process; 1 cm proximal to the iliac crest	Erector spinae muscles; quadratus lumborum (superficial); intraperitoneal space (deep to the muscle)	Transverse or longitudinal scan on posterior trunk	Avoid intraperitoneal space; inject only when muscle is clearly visualized
Rectus Femoris	Proximal third of anterior thigh; midline	Vastus intermedius (deep)	Transverse scan on anterior thigh	Avoid central tendon
Sartorius	Middle third of medial thigh	Adductor canal (deep)—femoral artery, vein, saphenous nerve, and nerve to vastus medialis; vastus medialis (deep and lateral) and adductor longus (deep and medial)	Transverse scan on medial thigh	Avoid structures in adductor canal
Gracilis	Proximal third of medial thigh	Adductor longus (deep and lateral), adductor magnus (deep), and semimembranosus (deep and medial)	Transverse scan with the hip abducted, externally rotated, and semi-flexed and the knee in semi-flexion	Visualize gracilis as most superficial medial muscle
Adductor Longus	Proximal third of medial thigh	Gracilis (medial), sartorius (lateral), adductor canal (lateral), and adductor brevis (deep)	Transverse scan on upper medial thigh	Confirm muscle position and relations, avoid structures in adductor canal
Adductor Magnus	Proximal third of medial thigh	Gracilis (superficial) and semimembranosus (medial)	Transverse scan with the hip abducted, externally rotated, and semi-flexed and the knee in semi-flexion	Identify deep muscle mass below gracilis
Semimembranosus	Distal third of posterior medial thigh	Semitendinosus (lateral), adductor magnus (deep and medial), sciatic nerve (deep and lateral), and popliteal bundle (deep)	Transverse scan on posterior medial thigh	Avoid neurovascular structures in distal thigh
Semitendinosus	Proximal third of posterior medial thigh	Semimembranosus (medial), biceps femoris (lateral), adductor magnus (deep), and sciatic nerve (deep and lateral)	Transverse scan on posterior thigh	Visualize characteristic ’Mercedes-Benz’ and ’Venetian blind’ signs, avoid nerve injury
Biceps Femoris	Distal third of posterior-lateral thigh (short head); mid-thigh for long head	Sciatic nerve (under long head)	Transverse scan at two levels on posterior thigh	Identify and differentiate long and short heads and avoid nerve injury

## Supplementary Materials

The following supporting information can be downloaded at: https://doi.org/10.5281/zenodo.15126622, Video S1: dynamic ultrasound evaluation of the *rectus femoris* (RF) muscle; Video S2: dynamic ultrasound evaluation of the *adductor longus* (AL) muscle; Video S3: dynamic ultrasound evaluation of the *semitendinosus* (ST) muscle.

## Abbreviations

The following abbreviations are used in this manuscript:

Ace	Acetabulum
AM	*Adductor magnus* (muscle)
AB	*Adductor brevis* (muscle)
AL	*Adductor longus* (muscle)
ASIS	Anterior superior iliac spine
a	artery
BF	*Biceps femoris* (muscle)
BFs	*Biceps femoris* (muscle) short head
BFI	*Biceps femoris* (muscle) long head
BoNT-A	Botulinum toxin type-A
CO	Coccyx (bone)
EUH	Elias University Hospital
ES	*Erector spinae* (muscle)
FV	Femoral (vein)
F	Femur (bone)
Gmax	*Gluteus maximus* (muscle)
Gmed	*Gluteus medius* (muscle)
Gmin	*Gluteus minimus* (muscle)
G	*Gracilis* (muscle)
GT	Greater trochanter (bone)
I	Iliac (bone)
IMF	Intramuscular fascia
IT	Ischial tuberosity
NtVM	(Nerve) to vastus medialis muscle
PSIS	Posterior superior iliac spine
PM	*Psoas major* (muscle)
QL	*Quadratus lumborum* (muscle)
RF	*Rectus femoris* (muscle)
SN	Saphenous (nerve)
Sart	*Sartorius* (muscle)
S	Sciatic (nerve)
SM	*Semimembranosus* (muscle)
ST	*Semitendinosus* (muscle)
VM	Vastus medialis (muscle)
v	vein
